# The relation of C - reactive protein to chronic kidney disease in African Americans: the Jackson Heart Study

**DOI:** 10.1186/1471-2369-11-1

**Published:** 2010-01-15

**Authors:** Ervin R Fox, Emelia J Benjamin, Daniel F Sarpong, Harsha Nagarajarao, Jason K Taylor, Michael W Steffes, Abdullah K Salahudeen, Michael F Flessner, Ermeg L Akylbekova, Caroline S Fox, Robert J Garrison, Herman A Taylor

**Affiliations:** 1 Department of Medicine, University of Mississippi School of Medicine, Jackson, MS, USA; 2 Department of Medicine, Boston University School of Medicine and School of Public Health, Boston, MA, USA; 3 Jackson Heart Study, Jackson State University, Jackson, MS, USA; 4 Department of Laboratory Medicine and Pathology, University of Minnesota, Minneapolis, MN, USA; 5 Section of Nephrology, Department of General Internal Medicine, University of Texas M. D. Anderson Cancer Center, Houston, TX, USA; 6 National Heart, Lung, and Blood Institute, National Institutes of Health, Bethesda, MD, USA

## Abstract

**Background:**

African Americans have an increased incidence and worse prognosis with chronic kidney disease (CKD - estimated glomerular filtration rate [eGFR] <60 ml/min/1.73 m^2^) than their counterparts of European-descent. Inflammation has been related to renal disease in non-Hispanic whites, but there are limited data on the role of inflammation in renal dysfunction in African Americans in the community.

**Methods:**

We examined the cross-sectional relation of log transformed C-reactive protein (CRP) to renal function (eGFR by Modification of Diet and Renal Disease equation) in African American participants of the community-based Jackson Heart Study's first examination (2000 to 2004). We conducted multivariable linear regression relating CRP to eGFR adjusting for age, sex, body mass index, systolic and diastolic blood pressure, diabetes, total/HDL cholesterol, triglycerides, smoking, antihypertensive therapy, lipid lowering therapy, hormone replacement therapy, and prevalent cardiovascular disease events. In a secondary analysis we assessed the association of CRP with albuminuria (defined as albumin-to-creatinine ratio > 30 mg/g).

**Results:**

Participants (n = 4320, 63.2% women) had a mean age ± SD of 54.0 ± 12.8 years. The prevalence of CKD was 5.2% (n = 228 cases). In multivariable regression, CRP concentrations were higher in those with CKD compared to those without CKD (mean CRP 3.2 ± 1.1 mg/L vs. 2.4 ± 1.0 mg/L, respectively p < 0.0001). CRP was significantly associated with albuminuria in sex and age adjusted model however not in the multivariable adjusted model (p > 0.05).

**Conclusion:**

CRP was associated with CKD however not albuminuria in multivariable-adjusted analyses. The study of inflammation in the progression of renal disease in African Americans merits further investigation.

## Background

The incidence and mortality of chronic kidney disease (CKD) is disproportionately higher in African Americans compared to their white counterparts. In a 12-year follow up cohort study of 9,082 African American and white adults between 30-74 years of age, African Americans' risk of CKD was 2.7 times higher than that of whites [1]. Although high rates of hypertension, diabetes and obesity in the middle-aged African American population contribute in part to the racial difference, the disparities were not fully accounted for by risk factor burden [1].

Recent data support the concept that C-reactive protein (CRP) is associated with the prevalence, progression and prognosis of renal dysfunction in non-Hispanic whites. Increasing CRP concentrations have been related to both prevalent [2-5] and incident [6] CKD in this group. Also a10-ml/minute/1.73 m^2 ^lower estimate GFR [(eGFR) among persons with eGFR <60 ml/minute/1.73 m^2^)] has been associated with an incidence rate ratio of 1.29 (95% confidence interval: 1.06, 1.55) for cardiovascular mortality and a doubling of albuminuria has been associated with an incidence rate ratio of 1.06 (95% confidence interval: 1.04, 1.08) for cardiovascular mortality [7]. CRP concentrations association with albuminuria may be affected by ethnicity and sex and this may part of the clustering of cardiovascular risk factors and the higher incidence of cardiovascular disease observed in African Americans [8]. Despite the increased incidence and poor sequelae from CKD, and the existing research in non-Hispanic whites relating inflammation to CKD, there are limited data on the relation of inflammatory biomarkers to renal function and albuminuria in African Americans. We hypothesized that renal function and albuminuria are significantly related to systemic inflammation in African Americans after adjusting for traditional cardiovascular risk factors.

## Methods

### Study Cohort and Design

The Jackson Heart Study is a longitudinal community-based observational cohort that was initiated in 2000 to prospectively investigate the epidemiology and determinants of cardiovascular disease in African Americans [9]. Thirty percent of study participants were former members of the Jackson, Mississippi cohort of the Atherosclerosis Risk in Communities study, and had been recruited by random selection from the driver's license registry [10]. Among the remaining participants, 23% were recruited by random selection from the "Accudata" list (a commercial listing that represents the overall tri-county population). An additional 23% were members of a constrained volunteer sample, in which recruitment was distributed among defined demographic cells in proportions designed to mirror those in the overall tri-county population. Twenty-four percent of participants were recruited through the Jackson Heart Study Family Study, as described [11]. Among the 5,301 participants recruited for Examination 1, we studied 4320 participants after excluding participants for the following indications: missing CRP measurements (n = 105); those with self-reported malignancy, those using medications suggesting underlying rheumatologic or inflammatory disease, and those with a white blood cell count > 12 suggesting infection (n = 312); missing covariates (n = 523); eGFR <15 ml/min/1.73 m^2 ^(n = 20); and missing eGFR (n = 21). The Jackson Heart Study was approved by the University of Mississippi Medical Center Institutional Review Board and participants gave written informed consent.

### Measurement of Renal Function

Biochemical testing for serum creatinine was performed at the University of Mississippi Medical Center Laboratory Reading Center using a multi-point enzymatic spectrophotometric assay on a Vitros 950 Ortho-Clinical Diagnostics analyzer. Creatinine values were biochemically calibrated to Cleveland Clinic-equivalent Minnesota Beckman CX3 assay for analysis purposes. Thus, the primary measure of renal function was eGFR, as calculated by the Modification of Diet and Renal Disease (MDRD) equation [12]:

CKD was defined in the primary analysis as eGFR <60 ml/min/1.73 m^2 ^[13,14]. In a secondary analysis, CRP relation to albuminuria [defined as an urinary albumin-to-creatinine ratio (UACR) of > 30 mg/g on spot or 24-hour urine collection] was considered. A thorough discussion of the measurements for CKD and their relation to interview and medical data in the Jackson Heart Study has been previously published [15].

### CRP measurement

We measured CRP using immunoturbidimetric CRP-Latex assay from Kamiya Biomedical Company following manufacturer's high-sensitivity protocol. The inter-assay coefficients of variation on control samples repeated in each assay were 4.5% and 4.4% at CRP concentrations of 0.45 mg/L and 1.56 mg/L respectively. The reliability coefficient for masked quality control replicates was 0.95 for the CRP assay.

### Covariates

Body mass index (BMI) was calculated as the ratio of fasting weight toheight squared (kg/m^2^). Obesity was defined as a BMI ≥30 kg/m^2^. We defined hypertension based on Joint National Commission VII guidelines (≥140/90 mmHg, or reported use of antihypertensive medications within two weeks prior to the visit) [16]. Our definition for diabetes was based on the American Diabetes Association guidelines: participants were considered to have diabetes if they had one of the following: fasting serum glucose ≥126 mg/dL or use of diabetic medications within two weeks of the clinic visit, or history of physician-diagnosed diabetes [17]. Fasting serum total to high density lipoprotein (HDL) cholesterol and triglyceride concentrations were assessed with Roche enzymatic methods using a Cobras centrifuge analyzer (Hoffman-La Roche). Smoking status was defined as any participant who had smoked at least 400 cigarettes in their lifetime and was currently smoking at their baseline examination. Prevalent cardiovascular disease was defined as a stated history of physician-diagnosed myocardial infarction or stroke, electrocardiographic evidence of myocardial infarction, or history of a revascularization procedure (percutaneous transluminal coronary angioplasty or coronary artery bypass surgery).

### Statistical Analysis

We performed descriptive statistics to determine the mean, standard deviation (SD) and/or percentages for study participants' characteristics. Frequency distributions were used for categorical data. Descriptive statistics were used to determine the characteristics of participants according to dichotomized CRP concentration (using the lower 75^th ^percentile as cutoff). Descriptive statistics were also used to determine characteristics of Jackson Heart Study participants with and without CKD.

Since its distribution was skewed, CRP concentration was natural log-transformed for clinical correlates analyses [18]. By mean of general linear modeling (SAS PROC GLM) age- and sex-adjusted linear regression was used to assess the relation of CRP concentrations (log transformed) to renal function (CKD). Multivariable regression was used to assess the relation of CRP to CKD accounting for cardiovascular disease risk factors including age, sex, body mass index (BMI), systolic and diastolic blood pressure, diabetes, total/HDL cholesterol, triglycerides, smoking, antihypertensive therapy, lipid lowering therapy, hormone replacement therapy, and prevalent cardiovascular disease events. Effect modification test of age, sex, obesity (BMI<30 versus ≥ 30 kg/m^2^), prevalent CVD and prevalent MI were performed and the analysis was stratified based on significant effect modifiers at the significance level of 0.01.

In a secondary analysis, we investigated the relation of CRP to albuminuria (UACR) by multivariable linear regression adjusting the above mentioned covariates. The presence or absence of albuminuria served as the independent variable in the model. Analyses were conducted with SAS version 9.2 (SAS Institute, Cary, NC). A two-sided p-value < 0.01 was considered statistically significant.

## Results

Table 1 displays the clinical characteristics of the Jackson Heart Study by CRP (the lower 75^th ^percentile and upper 25^th ^percentile) for the overall study population, for those participants age < 60 years and for those participants age ≥ 60 years. The prevalence of CKD based on eGFR criteria alone was 5.2% (n = 226); mean eGFR among individuals with CKD was 45.9 ± 13.7 ml/min/1.73 m^2^. CKD was more prevalent in those participants > 60 years old and the mean CRP concentration was slightly higher in this group.

**Table 1:** Characteristics of Study Participants in the Lower 75th and Upper 25^th ^percentile of C-Reactive Protein Stratified by Age 60 years

	Overall		Age < 60 years		Age ≥ 60 years	
	L75^th^P CRP (< 5.7 mg/L) n = 3254	U25^th^P CRP (≥ 5.7 mg/L) n = 1066	L75^th^P CRP (< 5.7 mg/L) n = 2071	U25^th^P CRP (≥ 5.7 mg/L) n = 663	L75^th^P CRP (< 5.7 mg/L) n = 1183	U25^th^P CRP (≥ 5.7 mg/L) n = 403
C-reactive protein mg/L	2.1 ± 1.5	14.4 ± 15.2	2.0 ± 1.5	14.1 ± 10.6	2.2 ± 1.5	14.9 ± 20.6
***Demographic factors***						
Age, years	54 ± 13	54 ± 12	46 ± 9	47 ± 8	67 ± 6	67 ± 6
Male, %	42.01	18.57	45.3	18.4	36.3	18.9
***Cardiovascular risk factors***						
Body mass index, kg/m^2^	30.3 ± 6.1	36.1 ± 8.3	30.4 ± 6.3	37.4 ± 8.3	30.1 ± 5.8	34.0 ± 7.8
Systolic blood pressure, mmHg	127 ± 18	127 ± 18	123 ± 16	124 ± 16	134 ± 20	133 ± 20
Diastolic blood pressure, mmHg	80 ± 10	78 ± 10	81 ± 10	80 ± 10	78 ± 10	75 ± 11
Hypertension, %	57.6	69.0	46.3	57.6	77.4	87.6
Diabetes, %	13.7	18.9	9.8	15.5	20.6	24.3
Total/HDL cholesterol, ratio	4.1 ± 1.3	4.1 ± 1.4	4.2 ± 1.4	4.1 ± 1.5	4.0 ± 1.2	4.1 ± 1.3
Triglyceride, mg/dL	104 ± 76	113 ± 80	102 ± 83	109 ± 69	107 ± 63	119 ± 95
Current smoker, %	12.2	14.4	14.3	15.9	8.7	12.0
Anti-hypertension therapy, %	46.8	60.1	34.3	48.9	68.6	78.5
Lipid lowering therapy, %	12.0	10.5	6.8	7.8	21.1	14.9
HRT use in women, %	11.2	24.1	10.6	22.9	12.1	26.0
***Nutritional factors***						
Total protein	79 ± 47	77 ± 48	86 ± 51	86 ± 53	66 ± 35	64 ± 35
***Kidney disease factors***						
Chronic kidney disease, %	4.2	8.4	1.5	4.2	8.9	15.4

Values are % or mean ± standard deviation

HRT, hormone replacement therapy, L75th = lower 75^th ^percentile, U25 = upper 25^th ^percentile

Chronic kidney disease is defined as a glomerular filtraction rate, 60 ml/min/1.73 m^2^

Table 2 shows the relation of CRP with CKD in the study population. There was evidence of effect modification by sex (p = 0.0061) therefore the analysis was stratified. For both men and women, CRP concentrations were higher in those with CKD compared to those without CKD. Between the CKD and non-CKD groups, there were greater differences in CRP in men compared to women. Therefore, the changes were in the same direction for both sexes, and the type of effect modification by sex was synergist. There was no effect modification by age, obesity, prevalent CVD or prevalent MI. Age-adjusted CRP concentration was significantly associated with CKD for both women (p = 0.02) and men (< 0.0001). After adjusting for age, BMI, systolic and diastolic blood pressure, diabetes, total/HDL cholesterol, triglycerides, smoking, lipid lowering therapy, hormone replacement therapy in women, and prevalent CVD events in the multivariable regression, CRP remained strongly associated with CKD for both women and men (p < 0.0001 for both). (See Additional file 1 for results of additional sub-analysis).

**Table 2:** Association of C-Reactive Protein with Chronic Kidney Disease (ml/min/1.73 m^2^) in the Pooled Study Population and Stratified by Sex

	CKD (eGFR < 60)	No CKD (eGFR ≥ 60)	p-value
Pooled**			
N	228	4092	
	CRP (Geometric Mean ± SE)	CRP (Geometric Mean ± SE)	
Adjustment			
Age and Sex	3.4 ± 1.1 mg/L	2.4 ± 1.0 mg/L	< 0.0001
Multivariable*	3.2 ± 1.1 mg/L	2.4 ± 1.0 mg/L	< 0.0001
			
**Women**			
N	178	2577	
	CRP (Geometric Mean ± SE)	CRP (Geometric Mean ± SE)	
Adjustment			
Age	3.9 ± 1.1 mg/L	3.0 ± 1.0 mg/L	0.016
Multivariable*†	3.7 ± 1.1 mg/L	3.1 ± 1.0 mg/L	< 0.0001
			
**Men**			
N	50	1515	
	CRP (Geometric Mean ± SE)	CRP (Geometric Mean ± SE)	
Adjustment			
Age	3.4 ± 1.2 mg/L	1.58 ± 1.0 mg/L	< 0.0001
Multivariable*†	3.1 ± 1.2 mg/L	1.58 ± 1.0 mg/L	< 0.0001

* adjusted for age, sex, body mass index, systolic and diastolic blood pressures, diabetes, current smoking status, hypertension drugs, lipid lowering drugs, hormone therapy replacement, triglycerides, total cholesterol/HDL ratio, and prevalent cardiovascular disease events.

** pooled signify both men and women included in the analysis

† sex is excluded from list of covariates since analysis is stratified by sex

CKD, Chronic kidney disease; eGFR, estimated glomerular filtration rate; CRP, C-reactive protein

### Secondary analyses

In a secondary analysis we assessed the relation of CRP with albuminuria (based on spot or 24-hour urine values with an UACR > 30 mg/g). (Table 3) The prevalence of albuminuria was 10.8% (n = 298) among the 2,750 participants with urine to calculate UACR. CRP was significantly associated with albuminuria in the age and sex adjusted model (< 0.0001) but was not in the multivariable-adjusted model (p = 0.16). There was no evidence of effect modification by age, sex, obesity, prevalent CVD or prevalent MI on the relation of CRP with albuminuria.

**Table 3:** Association of C-Reactive Protein with Albuminuria (UACR > 30 mg/g)

	Albuminuria Present	Albuminuria Absent	p-value
Pooled**			
N	298	2452	
	CRP (Geometric Mean ± SE)	CRP (Geometric Mean ± SE)	
Adjustment			
Age and Sex	2.2 ± 1.0 mg/L	3.0 ± 1.1 mg/L	< 0.0001
Multivariable*	2.3 ± 1.0 mg/L	2.5 ± 1.1 mg/L	0.16

* adjusted for age, sex, body mass index, systolic and diastolic blood pressures, diabetes,

current smoking status, hypertension drugs, lipid lowering drugs, hormone therapy replacement,

triglycerides, total cholesterol/HDL ratio, and prevalent cardiovascular disease events.

** pooled signify both men and women included in the analysis

UACR = urinary albumin-to-creatinine ratio; CRP, C-reactive protein

## Discussion

### Principal Findings

We found a significant association between higher mean CRP concentrations and CKD for women and men after adjusting for multiple traditional cardiovascular risk factors. In the secondary analysis, we found that CRP was significantly associated with albuminuria in age- and sex- adjusted models. CRP was not significantly associated with albuminuria in the multivariable adjusted model. This finding speaks against a glomerular involvement.

We found that the prevalence of CKD using eGFR by MDRD formula was 5.2% in this cohort. This result is somewhat surprising given the prevalence of stage III CKD was estimated at 7.7% in the recent analysis by Coresh et.al in the NHANES 1999-2004 population (a nationally representative sample of non-institutionalized adults aged 20 years or older) [15,19]. This may be affected by a number of factors including that hypertension control in our cohort was greater than that of the AA in NHANES and that differences in sample recruitment between our cohort and that in NHANES may favor a lower percentage of unhealthy individuals and subsequently lower prevalence of CKD in the Jackson Heart Study.

### Relation of CRP to CKD: Comparison with Prior Literature

Our finding of a significant association between CRP (as a marker of systemic inflammation) and CKD in the African American population-based cohort of the Jackson Heart Study is similar to that seen in the Netherlands Study (a large population-based cohort consisting of 7,317 non-Hispanic white individuals without diabetes). In a cross-sectional analysis of the cohort, CRP was significantly associated with lower GFR in the multivariable model (OR 1.9;95% CI 1.3 to 2.9) [5]. Two recent smaller studies in individuals with diabetes showed a relation between inflammatory markers and CKD, though in neither study was there a significant association with CRP[20,21] In the Cardiovascular Health Study, a community-based cohort of elderly individuals, CRP and other markers of systemic inflammation remained associated with a rise in creatinine after adjusting for multiple clinical risk factors including race (follow up period of nine years for the original cohort and four years for the more recently recruited African American cohort). Similar results were found for decline in estimated GFR. The decline in eGFR was greater with increasing number of inflammatory or prothrombotic markers that were above the median [22].

In addition to its relation to the progression of renal disease, CRP has been a strong predictor of cardiovascular events in those with reduced renal function [12,23]. For example, in an 8-year follow-up of women in the Nurses' Health Study, the odds ratio per 5 mg/L increase in CRP was 1.68 (95% CI, 1.13 to 2.52) for women with an estimated creatinine clearance of ≤ 74 ml/min compared with 1.23 (95% CI, 0.86 to 1.76) and 0.99 (95% CI, 0.76 to 1.29) for women with an estimated creatinine clearance of 75 to 89 and ≥ 90 ml/min, respectively. Those with end-stage renal disease but not on dialysis and those with mild renal insufficiency are at increased risk for the development of cardiovascular disease, which cannot be attributed entirely to traditional risk factors of diabetes, hypertension and obesity [24,25]. Our results suggest that inflammation relates to CKD and may represent an additional (non-traditional) risk factor for cardiovascular events in those with CKD.

### Mechanism linking CRP with renal function

A number of theories have been advanced to explain the link between the deterioration of renal function and inflammation. One theory is that renal dysfunction is related to inflammation through the reduction in the nitric oxide synthase activity [26]. Nitric oxide synthase is linked to inflammation through its role in the formation of peroxynitrate, an important mediator of the immune response. One proposed explanation for higher CRP concentrations with CKD is that there is diminished filtration of CRP in end-stage renal disease [24]. However, it has been established that CRP is filtered in negligible amounts in the glomerulus and reabsorbed by the distal renal tubules. Therefore, elevation of CRP in CKD is not satisfactorily answered by this theory [24,27].

Yet another theory suggests that CKD and CRP are related through shared risk factors. It is known that hypertension, obesity and diabetes are all strongly related to both systemic inflammation and renal dysfunction [5]. Inflammation may be associated with hypertension and the development and progression of renal dysfunction through activation of the renin-angiotensin system and its influence on endothelial function [28-31]. Similarly inflammation is related to obesity through the production of cytokines by adipocytes [32,33] and may therefore be related to renal dysfunction through this relation with obesity. It is known that obesity is associated with end-stage renal disease through its strong correlation with type 2 diabetes and hypertension [5].

Finally, others have shown that higher CRP concentrations are related to reduced kidney cortex width and reduced renal cortex width is related to increased blood pressure [34,35] Hence, CRP may be a marker of kidney inflammation with increased scarring in the kidney cortex, which may then relate to blood pressure.

### Strengths and Limitations

There are a number of limitations of our study. First, the study population consists solely of African Americans and so the generalizability of this study to other ethnic groups is unclear. Second, this study is cross-sectional and therefore cause-effect relations cannot be determined. Third, because data are derived from the first visit for the Jackson Heart Study cohort, investigators were unable to assess the effects of longitudinal exposure to environmental factors. Fourth, renal function in the current analysis was estimated using the MDRD eGFR formula (a recommended means of estimating kidney function). MDRD has stronger specificity than sensitivity in predicting kidney problems. The more direct measure of kidney disease (using iothalamate or iohexol clearance to measure GFR) is not feasible in a population-based setting. Balanced against these caveats, the strength of this study lies in the prospective collection of CRP concentrations and covariates in a large population-based cohort of African Americans.

## Conclusion

Our study found that CRP is significantly related to CKD as defined by eGFR < 60 ml/min/1.73 m^2 ^in African Americans. Given the disproportionate burden of renal dysfunction in the African American community, future investigations should focus on the relation of CRP to the development and progression of renal disease and whether lower CRP in longitudinal studies predict improvement in renal function over time. Such findings would suggest that CRP as a marker of systemic inflammation may serve as a potential target for treatment and prevention. Research into genetic factors contributing to CRP variation in African Americans may also provide further understanding of how systemic inflammation relates to renal dysfunction in this group [36].

## Abbreviations

BMI: denotes body mass index; CKD: denotes chronic kidney disease; CRP: denotes C-reactive protein; CVD: denotes cardiovascular disease; eGFR: represents estimated glomerular filtration rate; MDRD: Modification of Diet in Renal Disease; UACR: denotes urinary albumin to creatinine ratio.

## Competing interests

The authors declare that they have no competing interests.

## Authors' contributions

EF conceived the study and participated in the design and drafted the manuscript. EB participated in the design of the study and revised the manuscript critically for important intellectual content, DA participated in the design of the study and performed the statistical analysis, HN and JT both participated in the design of the study and the drafting of the manuscript, MS performed assays on CRP and contributed to revising the manuscript for important intellectual content. AS, MF and CF each participated in the design and revised the manuscript critically for important intellectual content, EA derived key variables important for statistical analysis and participated in the design of the manuscript, RG made significant changes to the statistical analysis section and drafting of the manuscript and HT contributed by making critical revisions to the manuscript for intellectual content. All authors read and approved the final manuscript.

## Pre-publication history

The pre-publication history for this paper can be accessed here:

http://www.biomedcentral.com/1471-2369/11/1/prepub

## Supplementary Material

Additional file 1 **Summary Results of Additional Sub-analysis**. Additional sub-analysis was performed examining the association between CRP (log transformed) and CKD status by means of linear regression models. Two set of linear regression models were derived, the first was age and sex-adjusted and the second was multivariable adjusted. CKD status was categorized into normal (eGFR ≥ 90 and absence of albuminuria) and CKD Stages 1 (eGFR ≥ 90 and presence of albuminuria), 2 (60 ≤ eGFR ≤ 89 and presence of albuminuria), and 3 (30 ≤ eGFR ≤ 59). P trend was computed to assess the linear trend in the levels of CRP as one progress from normal to Stage 3 of CKD. Results of the analysis are summarized below (see Table S2a). The geometric means ( ± standard error) and confidence intervals for the various categories of CKD status are provided in a Table S2a. entitled "Association of C-Reactive Protein with Chronic Kidney Disease (ml/min/1.73 m^2^)". Click here for file
