# Supplementary material for: The relation of C - reactive protein to chronic kidney disease in African Americans: the Jackson Heart Study
Source: BMC Nephrol. 2010 Jan 15;11:1. doi: 10.1186/1471-2369-11-1 (PMC2826325; doi:10.1186/1471-2369-11-1)
Supplement: Additional file 1 — Summary Results of Additional Sub-analysis. Additional sub-analysis was performed examining the association between CRP (log transformed) and CKD status by means of linear regression models. Two set of linear regression models were derived, the first was age and sex-adjusted and the second was multivariable adjusted. CKD status was categorized into normal (eGFR ≥ 90 and absence of albuminuria) and CKD Stages 1 (eGFR ≥ 90 and presence of albuminuria), 2 (60 ≤ eGFR ≤ 89 and presence of albuminuria), and 3 (30 ≤ eGFR ≤ 59). P trend was computed to assess the linear trend in the levels of CRP as one progress from normal to Stage 3 of CKD. Results of the analysis are summarized below (see Table S2a). The geometric means ( ± standard error) and confidence intervals for the various categories of CKD status are provided in a Table S2a. entitled "Association of C-Reactive Protein with Chronic Kidney Disease (ml/min/1.73 m2)". [file 1471-2369-11-1-S1.DOC]

**Summary Results of Additional Sub-analysis**

Additional sub-analysis was performed examining the association between CRP (log transformed) and CKD status by means of linear regression models. Two set of linear regression models were derived, the first was age and sex-adjusted and the second was multivariable adjusted. CKD status was categorized into normal (eGFR ≥ 90 and absence of albuminuria) and CKD Stages 1 (eGFR ≥ 90 and presence of albuminuria), 2 (60 ≤ eGFR ≤ 89 and presence of albuminuria), and 3 (30 ≤ eGFR ≤ 59). P trend was computed to assess the linear trend in the levels of CRP as one progress from normal to Stage 3 of CKD. Results of the analysis are summarized below (see Table 2a). The geometric means (± standard error) and confidence intervals for the various categories of CKD status are provided in Table 2a.

**Table 2a. Association of C-Reactive Protein with Chronic Kidney Disease (ml/min/1.73 m^2^)**

|  |  | **Stages of CKD** | | | **ptrend** |
| --- | --- | --- | --- | --- | --- |
|  | Normal | 1 | 2 | 3 |  |
| Pooled** |  |  |  |  |  |
| N | 1068 | 120 | 2336 | 208 |  |
|  | CRP GM±SE  (95% CI) | CRP GM±SE  (95% CI) | CRP GM±SE  (95% CI) | CRP GM±SE  (95% CI) |  |
| Adjustment |  |  |  |  |  |
| Age and Sex | 2.3±1.0  (0.2, 4.3) | 3.6±1.1  (1.4, 5.8) | 2.4±1.0  (0.3, 4.4) | 3.5±1.1  (1.3, 5.6) | 0.0099 |
| Multivariable* | 2.4±1.0  (0.4, 4.5) | 2.9±1.1  (0.7, 5.1) | 2.3±1.0  (0.3, 4.3) | 3.1±1.1  (1.0, 5.3) | 0.0734 |
| * adjusted for age, sex, body mass index, systolic and diastolic blood pressures, diabetes, current smoking status, hypertension drugs, lipid lowering drugs, hormone therapy replacement, triglycerides, total cholesterol/HDL ratio, and prevalent cardiovascular disease events. ** pooled signify both men and women included in the analysis  CKD, Chronic kidney disease; eGFR, estimated glomerular filtration rate  GM = Geometric Mean and SE = Standard Error | | | | | |
